# Double jeopardy - pituitary apoplexy complicated by ruptured aneurysm of the internal carotid artery within an adenoma: a case report

**DOI:** 10.1186/s12883-022-02999-2

**Published:** 2022-12-09

**Authors:** Nutnicha Pattaravimonporn, Wasawat Muninthorn, Thanwa Sudsang, Ake Hansasuta, Dararat Chiewchalermsri, Chutintorn Sriphrapradang

**Affiliations:** 1 Department of Medicine, Faculty of Medicine Ramathibodi Hospital, Mahidol University, Bangkok, 10400 Thailand; 2 Department of Surgery, Division of Neurosurgery, Faculty of Medicine Ramathibodi Hospital, Mahidol University, Bangkok, 10400 Thailand; 3 Department of Diagnostic and Therapeutic Radiology, Faculty of Medicine Ramathibodi Hospital, Mahidol University, 10400 Bangkok, Thailand

**Keywords:** Aneurysm, Case report, Computed tomography, Headache, Haemorrhage, Magnetic resonance imaging, Pituitary neoplasms

## Abstract

**Background:**

Sudden onset of severe headache is the most common presentation of a ruptured intracranial aneurysm. Similar symptoms can be caused by pituitary apoplexy, and radiological examination is needed to distinguish between the two. Development of infarction and/or haemorrhage of the hypophysis with concomitant unruptured cerebral aneurysm has been described. However, intratumoural aneurysm within a pituitary adenoma presenting with the ictus of both pathologies is extremely rare.

**Case presentation:**

A 64-year-old man presented with classic symptoms of pituitary apoplexy. His symptoms improved after a few days, and he was discharged. However, he subsequently developed further episodes of sudden and severe headache together with visual decline and ophthalmoplegia. Radiographs demonstrated an enlarging sellar mass with suspicion of a ruptured internal carotid artery aneurysm, within the apoplectic pituitary macroadenoma. Although an endovascular procedure was planned, the patient developed massive subarachnoid haemorrhage resulting in brain death.

**Conclusion:**

This case report describes an intratumoural aneurysm of the cavernous internal carotid artery as a potential cause or result of pituitary apoplexy. Despite its rarity, this possible life-threatening complication of pituitary apoplexy should be recognised for prompt diagnosis and early management.

## Background

Pituitary apoplexy is characterised by infarction and/or haemorrhage of the pituitary gland or adenoma. Patients typically present with abrupt onset of severe headache, visual disturbance, and hypopituitarism, which may be life-threatening [1]. Radiographic imaging is needed to confirm the diagnosis and to rule out subarachnoid haemorrhage (SAH) from a ruptured intracranial aneurysm (IA). Whereas a ruptured IA and pituitary apoplexy are common as separate conditions, the occurrence of both pathologies in the same episode is extremely rare and has been described in only a few reports [2–4]. We herein describe a patient with an initial presentation of pituitary apoplexy that was subsequently complicated by rupture of an intratumoural aneurysm of the internal carotid artery (ICA).

## Case presentation

**Fig. 1:**
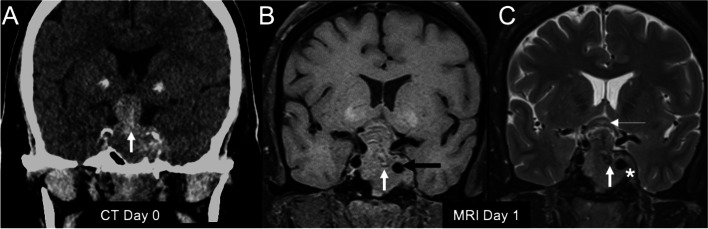
Initial imaging examinations of the 64-year-old man obtained from the outside hospital. **A** An unenhanced computed tomography image showed the typical “snowman” or “figure of 8” configuration of pituitary macroadenoma with internal hyperdense areas consistent with acute haemorrhage within the tumour (white arrow). **B**-**C** This haemorrhagic macroadenoma was confirmed on magnetic resonance images obtained 1 day later. The foci of haemorrhage were isointense to slightly hyperintense (white arrow) on unenhanced T1-weighted imaging (**B**) and hypointense (white arrow) on T2-weighted imaging (**C**). The tumour was found to be encasing the normally sized left cavernous internal carotid artery (black arrow in (**B**)) and invading the left cavernous sinus (asterisk in (**C**)). **C** The optic chiasm (thin white arrow) was elevated by the suprasellar part of the tumour

**Fig. 2:**
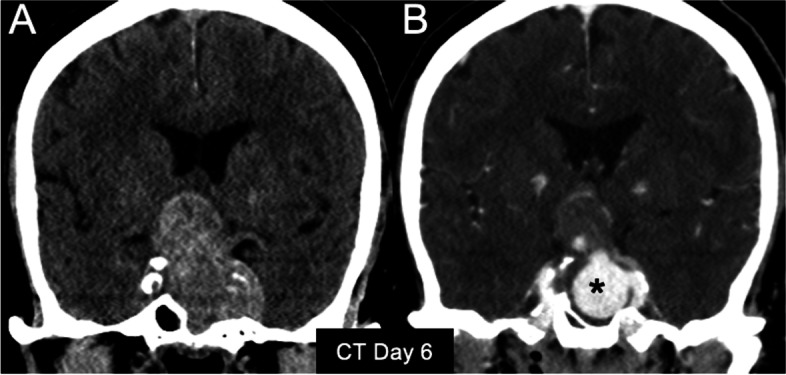
**A** Unenhanced and (**B**) enhanced computed tomography (CT) images obtained from the outside hospital soon after the patient experienced the second attack of severe headache. **B** A vividly enhancing pouch (asterisk) on contrast-enhanced CT imaging appeared to communicate with the left cavernous internal carotid artery. The findings were highly suggestive of intratumoural pseudoaneurysm formation, although the outside CT report indicated an enlarging apoplectic macroadenoma

A 64-year-old man presented with a sudden onset of severe headache and vomiting. According to the report from an outside hospital, his initial neurological examination was only remarkable for bitemporal hemianopia. A non-contrast computed tomography (CT) scan of the brain showed a pituitary adenoma (PA) with haemorrhage (Fig. 1A). Magnetic resonance imaging (MRI) the following day confirmed a 26- x 33- x 42-mm tumour with haemorrhagic apoplexy (Fig. 1B, C). This sizable macroadenoma significantly compressed the optic apparatus and invaded the left cavernous sinus. The left cavernous ICA was encased by the PA. The patient reported a 30-year history of loss of libido and erectile dysfunction. His family and psychosocial history was unremarkable. The initial hormonal evaluation at the local hospital had showed central hypothyroidism and hypogonadotropic hypogonadism; the prolactin level was pending. Hormonal replacement was started for his panhypopituitarism before hospital discharge. Four days later, while recovering from the first onset of headache, the patient developed another episode of sudden headache. During this second episode, he developed blindness of both eyes and became more somnolent. Physical examination showed left-sided ptosis, ophthalmoplegia, and a relative afferent pupillary defect in the left eye. Fundoscopic examination demonstrated bilateral pale optic discs. Emergency brain CT with and without contrast showed enlargement of the PA with apoplexy as interpreted by a local radiologist (Fig. 2A, B).

**Fig. 3:**
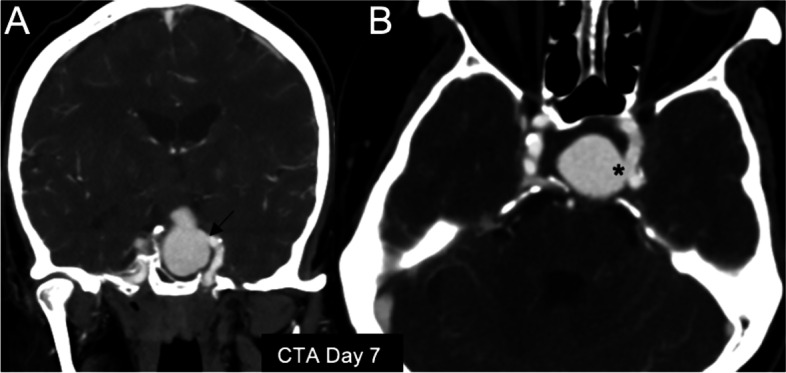
Computed tomography angiography images obtained at our hospital clearly demonstrated an intratumoural pseudoaneurysm originating from the left cavernous internal carotid artery. **A** Coronal reconstruction image and (**B**) axial reconstruction image showed an early enhancing pouch directly communicating with the left cavernous internal carotid artery (black arrow in (**A**), asterisk in (**B**))

Because of the lack of equipment at the local facility, the patient was transferred to our hospital for a transsphenoidal procedure 7 days after the first onset of headache. Because of the peculiar enhancement noted in the contrasted CT scan from the referring hospital, an ICA aneurysm was indeed probable. Hence, CT angiography was performed shortly after his arrival at our hospital. This imaging examination demonstrated a 22- x 23- x 30-mm lobulated arterial-enhancing outpouching lesion within a 26- x 34- x 46-mm apoplectic PA (Fig. 3A, B). The radiographic features were consistent with a pseudoaneurysm communicating with the medial aspect of the left cavernous ICA. The patient had no risk factors for cavernous ICA, such as prior trauma. On the day of his arrival at our hospital, his prolactin level that had been obtained at the referring hospital was found to be 811.02 ng/mL (reference range, 3.46–19.40 ng/mL). However, a dopamine agonist had not been started by the local hospital at the time of blood sample collection.

**Fig. 4:**
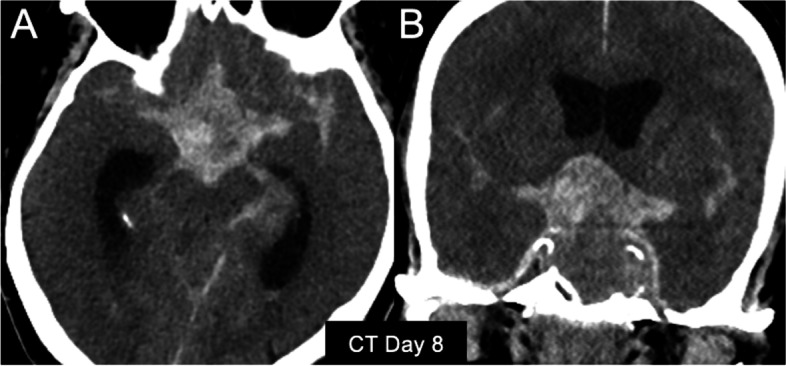
Unenhanced computed tomography images obtained immediately after the third attack of severe headache. **A** Axial and (**B**) coronal images showed massive subarachnoid haemorrhage distributed in the suprasellar cistern, bilateral sylvian fissures, perimesencephalic cistern, and ventricular system. Haemorrhage was present within the contour of the tumour. Hydrocephalus was noted

We were concerned that the intratumoural pseudoaneurysm would complicate our intended transsphenoidal access for PA resection. Therefore, we consulted with the endovascular team. Unfortunately, while arranging for neurovascular intervention, the patient developed a third attack of sudden headache and became comatose. A subsequent CT scan revealed massive, newly developed SAH. Worsening of both the irregular contour and the internal density within the PA was also observed (Fig. 4A, B). Re-rupture of his pseudoaneurysm was the most likely aetiology. His family did not wish to pursue aggressive treatment for his moribund status and declined a post-mortem examination.

## Discussion and conclusion

With the recent advances in imaging technologies for preoperative detection, the incidence of concomitant PA with an unruptured IA has ranged from 2.3 to 8.3% in the last decade [5, 6]. Although it is possible for such patients to develop either apoplexy from PA or SAH from a ruptured IA, the simultaneous ictus of both pathologies is extremely rare. Yoshida et al. [4] recently described a patient who developed SAH from an anterior communicating artery aneurysm. Within a few days, the patient developed progressively declining vision, most likely due to the aneurysm embedding within the PA. The authors also reviewed four other publications of similar events. All of the cited case reports of SAH associated with pituitary apoplexy were due to ruptured aneurysms at the supraclinoid parts of the ICA [4]. A recent systematic review by Piper et al. [7] selected only case reports in which the ICA aneurysms had direct contact with the co-existing PAs. Most of them were unruptured aneurysms at the cavernous ICA. The review showed that only 2 of 21 patients with PA had aneurysm rupture that caused massive epistaxis, but none had SAH [7].

Our present case involved an exceedingly rare occurrence of a ruptured intratumoural ICA aneurysm that initially caused severe headache and visual decline from pituitary apoplexy. A patient with a similar condition was described by Suzuki et al. [2]. This patient also had a high serum prolactin level but had no SAH. Unlike our patient, whose treatment was delayed by the interhospital transfer process, the patient described by Suzuki et al. [2], underwent urgent transsphenoidal surgery for tumour resection. Short of a preoperative vascular imaging study, the surgeons unexpectedly came across massive bleeding during the procedure. After significant intraoperative haemorrhage, temporary tamponade finally achieved haemostasis, and subsequent cerebral angiography revealed a cavernous ICA aneurysm. Eventually, endovascular occlusion of the ICA was performed with a good outcome [2]. Krug et al. [3] reported another similar case involving a patient who had Crooke cell pituitary apoplexy with a simultaneous 3-mm intratumoural pseudoaneurysm of the ICA and normal ophthalmologic examination findings. This patient underwent uneventful partial tumour resection with intentional sparing of the vicinity of the pseudoaneurysm via the transsphenoidal route, and a flow-diverting stent was placed thereafter [3]. In retrospect, based on the two aforementioned case reports in which the patients survived, our patient could have been more ideally cared for by 1) undergoing CT angiography at the local hospital to save time before his transfer to our institute; 2) undergoing an endovascular procedure to assess the collateral circulation and determine if complete occlusion of the left ICA was necessary; and 3) after the aneurysm had been secured, undergoing transsphenoidal surgery or receiving a dopamine agonist as early as possible. A hybrid operating theatre could have also been helpful in this situation. However, with the delays of the time-consuming inter-hospital transfer and the repeated rupture of the aneurysm, the patient eventually died. Despite the high index of suspicion of a pseudoaneurysm on CT angiography, a pseudoaneurysm was not confirmed either by cerebral angiography or at autopsy.

Unlike a true aneurysm, which is bounded by all three layers of the arterial wall (tunica intima, tunica media, and tunica adventitia), a pseudoaneurysm, or false aneurysm, is an abnormal outpouching or dilation of an artery that is only bounded by the outermost layer of the arterial wall (tunica adventitia). Because of the poor support of the pseudoaneurysm wall, it is unstable and thus poses a higher risk of rupture than a true aneurysm of comparable size. Additionally, there is a risk of growth and rupture due to the pulsatile inflow and outflow of blood through the neck of the pseudoaneurysm. Although rare, death may occur if an intracranial pseudoaneurysm remains untreated [8]. The causative effect of a pseudoaneurysm on the development of pituitary apoplexy, or vice versa, is debatable. In the present case, the small area of blood medial to the left cavernous ICA on the first CT scan could have represented early haemorrhage from either the pseudoaneurysm or the apoplexy. However, in contrast to typical patients with apoplexy, our patient experienced multiple episodes of increasing severity within a very short time span; this is largely uncommon for apoplectic PA. What exactly triggered this catastrophic event remains unknown. In this case, the rapid expansion of apoplectic PA could have led to vascular injuries, potentially resulting in pseudoaneurysm formation. Atherosclerosis, as evidenced by imaging, may jointly promote pseudoaneurysm development. However, the true aetiology of the pseudoaneurysm in this case is unknown. As a result, we acknowledge that this case report has some limitations.

In conclusion, we have herein described the development of an intratumoural pseudoaneurysm of the cavernous ICA as a potential cause or result of pituitary apoplexy. The possibility of a complicated aneurysm should be considered in apoplectic patients with recurring episodes of severe headache with a crescendo pattern and a mass effect to the surrounding structures.

## Data Availability

All data related to this case report are documented within this manuscript.

## Abbreviations

CT: Computed tomography
IA: Intracranial aneurysm
ICA: Internal carotid artery
MRI: Magnetic resonance imaging
PA: Pituitary adenoma
SAH: Subarachnoid haemorrhage
